# Identification and Expressional Analysis of siRNAs Responsive to *Fusarium graminearum* Infection in Wheat

**DOI:** 10.3390/ijms242116005

**Published:** 2023-11-06

**Authors:** Kai Fu, Qianhui Wu, Ning Jiang, Sijia Hu, Hongyan Ye, Yi Hu, Lei Li, Tao Li, Zhengxi Sun

**Affiliations:** 1 Jiangsu Key Laboratory of Crop Genomics and Molecular Breeding, Key Laboratory of Plant Functional Genomics of the Ministry of Education, Jiangsu Key Laboratory of Crop Genetics and Physiology, Agricultural College of Yangzhou University, Yangzhou 225009, China; 211702501@stu.yzu.edu.cn (K.F.); 211702520@stu.yzu.edu.cn (Q.W.); mz120211290@stu.yzu.edu.cn (N.J.); s20223010048@cau.edu.cn (S.H.); mz120221311@stu.yzu.edu.cn (H.Y.); dx120220130@stu.yzu.edu.cn (Y.H.); lilei@yzu.edu (L.L.); taoli@yzu.edu.cn (T.L.); 2 Jiangsu Co-Innovation Center for Modern Production Technology of Grain Crops, Yangzhou University, Yangzhou 225009, China

**Keywords:** small RNA deep sequencing, 24 nt siRNAs, *TaDCL3*, RNA-directed DNA methylation, FHB-resistance

## Abstract

The outbreak of Fusarium head blight (FHB) poses a serious threat to wheat production as it leads to both significant yield losses and accumulation of several mycotoxins including deoxynivalenol (DON) in the grains, which are harmful to human and livestock. To date, hundreds of FHB-resistance-related quantitative trait loci (QTLs) have been reported, but only a few of them have been cloned and used for breeding. Small interfering RNAs (siRNA) have been reported in plants to mediate host defense against pathogens, but they have rarely been reported in wheat-FHB interaction. In order to identify the key siRNAs that can potentially be used in the improvement of resistance to FHB, siRNAs from the spikes of an FHB-resistant variety Sumai 3 and an FHB-susceptible variety of Chinese Spring (CS) were sequenced after *F. graminearum* infection and mock inoculation, respectively. The expression patterns of the siRNAs of interest were analyzed. A total of 4019 siRNAs of high-confidence were identified, with 131 being CS-specific, 309 Sumai 3-specific and 3071 being common in both varieties. More than 87% of these siRNAs were 24 nt in length. An overall down-regulation trend was found for siRNAs in the spikes of both varieties after being infected with *F. graminearum*. The expression patterns for *Triticum aestivum* Dicer-like 3 (*TaDCL3*) that synthesizes 24 nt siRNAs were validated by qRT-PCR, which were positively correlated with those of the siRNAs. A total of 85% of the differentially expressed genes putatively targeted by the siRNAs were significantly up-regulated after infection, showing a negative correlation with the overall down-regulated expression of siRNAs. Interestingly, the majority of the up-regulated genes are annotated as disease resistance. These results suggested that the inhibition of siRNA by *F. graminearum* up-regulated the disease resistance genes, which were putatively suppressed by siRNAs through RNA-directed DNA methylation (RdDM). Consequently, the resistant capability to *F. graminearum* infection was enhanced. This study provides novel clues for investigating the function of siRNA in wheat-*F. graminearum* interaction.

## 1. Introduction

Fusarium head blight (FHB) is one of the most serious fungal diseases in wheat, and the *Fusarium graminearum* species complex is considered to be the main pathogen in FHB [1]. FHB epidemics result in a significant decrease in wheat yield and mycotoxin contamination in wheat grains. Consumption of these contaminated grains leads to decreased immunity and gastrointestinal diseases in both humans and animals [2]. FHB is a disease that frequently occurs in humid areas. High temperature and humidity conditions promote the germination of *F. graminearum* spores and infection incidence [3]. Wheat can be infected by *F. graminearum* at the stages from heading to maturity, and the anthesis stage is the most susceptible stage [1]. The anther exposure rate is correlated with wheat susceptibility to FHB [4]. FHB epidemics in wheat take place sporadically all over the world. However, the frequency of FHB epidemics has increased in the last decades in China. In the past two decades, the disease has spread northward to the major production area of the Yellow and Huai River Valleys from the middle and lower reaches of the Yangtze River. In addition, the content of DONs in the crops have been significantly increased under the corn–wheat rotation system [5]. Therefore, there is an urgent need to control wheat FHB.

At present, cultivating varieties with stable genetic resistance using modern molecular breeding techniques is considered to be the fundamental approach to solve the danger of FHB [6]. Wheat resistance to FHB is considered as a quantitative trait controlled by multiple genes, and hundreds of the quantitative trait loci (QTL) related to FHB resistance have been mapped [7,8,9]. *Fhb1* is the most important QTL, which is derived from the resistant wheat variety Sumai 3 and provides stable resistance to FHB [10,11]. Although *Fhb1* is the major QTL for FHB resistance, previous studies have revealed that genetic backgrounds influence the expression of *Fhb1* [12]. Therefore, mining new resistance-related genes is beneficial to the genetic improvement and control of wheat FHB.

Small RNAs in plants play an important role in regulating gene expression. They are classified into three categories, namely microRNA (miRNA), short interfering RNA (siRNA) and tRNA derived fragment (tRF) according to their source and functions [13,14]. siRNA refers to a type of short non-coding RNA that is derived not only from the endogenous plant genome but also from the exogenous genomes, including microbes or foreign plant genes introduced by transgenes [15].Double-stranded precursors of siRNA are usually synthesized from long double-stranded RNA (dsRNA) preformed by the RNA-dependent RNA polymerase (RDR) pathway [16,17]. Biosynthesis of mature siRNA from longer dsRNA molecules depends on Dicer-like (DCL) endoribonuclease. Short dsRNA strands generated by DCL are loaded into Argonaute proteins (AGOs), which target endogenous or exogenous RNAs based on sequence complementarity to mediate gene or transposon silence [18]. Four proteins of the DCL family were found to cleave stem-loop or double-stranded (ds) RNA precursors into miRNAs or siRNAs of specific sizes. DCL1 mainly plays a major role in the formation of miRNA, and miRNA regulates the post-transcriptional level of target genes by mediating target gene mRNA cleavage or translational repression [19]. DCL2 was found to regulate the synthesis of 22 nt siRNA, which can trigger plant-specific and systemic gene silencing and translational repression [20]. DCL3 cleaves dsRNA precursors to form 24 nt siRNAs, which mainly regulate transcriptional repression of genes via RNA-directed DNA methylation (RdDM) [21]. DCL4 is an essential nuclease in the formation of 21 nt siRNAs, which mediate the cleavage of target genes and exert post-transcriptional regulatory functions similar to miRNAs [22]. Plants are subjected to various stresses during their growth and development, especially the stresses of pathogenic microorganisms. Therefore, plants have evolved complex gene silencing mechanisms to resist the invasion of various pathogens. The first siRNA reported to regulate plant immunity is nat-siRNAATGB2, which is highly specifically induced by the effector *avrRpt2* and carried by the bacterial pathogen *Pseudomonas syringae*. nat-siRNAATGB2 silencing a negative pentapeptide repeat protein contributes to disease resistance of plant [23]. In addition, siRNA can also perform the function of RNA interference (RNAi) via the cross-kingdoms pathway, and host-derived small RNAs are delivered to pathogens through extracellular vesicles and inhibit the expression of virulent genes in fungal pathogens [24,25]. So far, there has been no report about siRNA regulating wheat resistance to FHB.

In this study, RNA sequencing was performed on an FHB-susceptible variety of Chinese Spring (CS) and an FHB-resistant variety Sumai 3 (SM) after *F. graminearum* infection and mock inoculation, respectively, to identify siRNAs involved in host–pathogen interaction. The differential expression patterns of the identified siRNAs revealed that the majority of the siRNAs were inhibited by *F. graminearum*. We also analyzed the potential regulatory mechanism of siRNA for wheat response to FHB. This study provides new clues for further research on the biological function of siRNAs in wheat, and also provides a new perspective on breeding for wheat resistance to FHB.

## 2. Results

### 2.1. Identification of siRNAs in Wheat Spike via Small RNA Deep Sequencing

**Figure 1:**
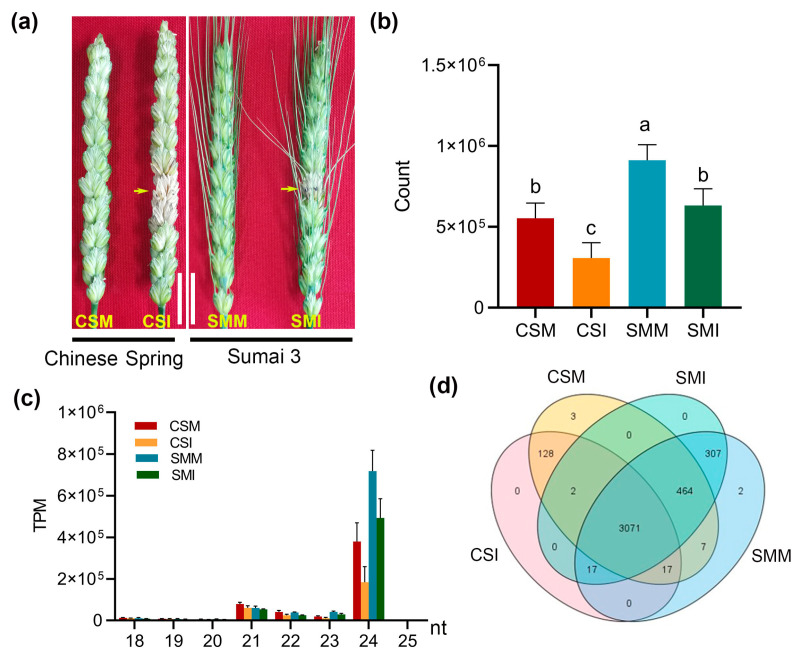
Overview of sRNA in CS and SM after infection with *F. graminearum*. (**a**) Symptoms of CS and SM after *F. graminearum* infection. The picture shows the state of the spikes photographed 7 days post infection with *F. graminearum*. Yellow arrows indicate spikelets infected with *F. graminearum*. CSM, CS inoculated with mock (mung bean soup); CSI, CS infected with *F. graminearum*; SMM, SM inoculated with mock (mung bean soup); SMI, SM infected with *F. graminearum*. The scale bar = 2 cm. (**b**) Total count of siRNA in different groups. The calculated value is the mean ± standard error of the three bioreplicates. Differences between groups were analyzed using Student–Newman–Keulsa multiple test, and different letters indicate different levels of difference (*p* < 0.05). (**c**) Length distribution of siRNA. nt, nucleotide. (**d**) Venn distribution of the identified siRNAs in the four libraries.

There were significant differences in the symptoms of wheat spikes infected with *F. graminearum* between CS and SM. Seven days after infection, 3–4 symptomatic spikelets appeared on the spike of CS, but only one symptomatic spikelet appeared on the spikes of SM (Figure 1a). Compared with the control (mock inoculation), the overall siRNA expressions in CS and SM were significantly reduced after *F. graminearum* infection. The overall siRNA expression level in SM was significantly higher than that in CS regardless of *F. graminearum* infection or mock inoculation (Figure 1b). The siRNA lengths were mainly distributed between 21 nt and 24 nt, among which 24 nt siRNA had the highest expression level, accounting for 87% of the total siRNA (Figure 1c). A total of 7027 putative siRNAs were identified by small RNA deep sequencing. Among these putative siRNAs, the siRNAs of low-confidence and the siRNAs with an average expression value less than 50 TPM in the four library sets were removed, resulting in a total of 4019 high-confidence siRNAs. There were 3071 siRNAs co-expressed in the CSM, CSI, SMM and SMI groups (Table S1), and 131 specific siRNAs were detected in the CS; 309 specific siRNAs were detected in SM (Figure 1d).

### 2.2. Most of siRNAs Were Down-Regulated after Infection with F. graminearum

**Figure 2:**
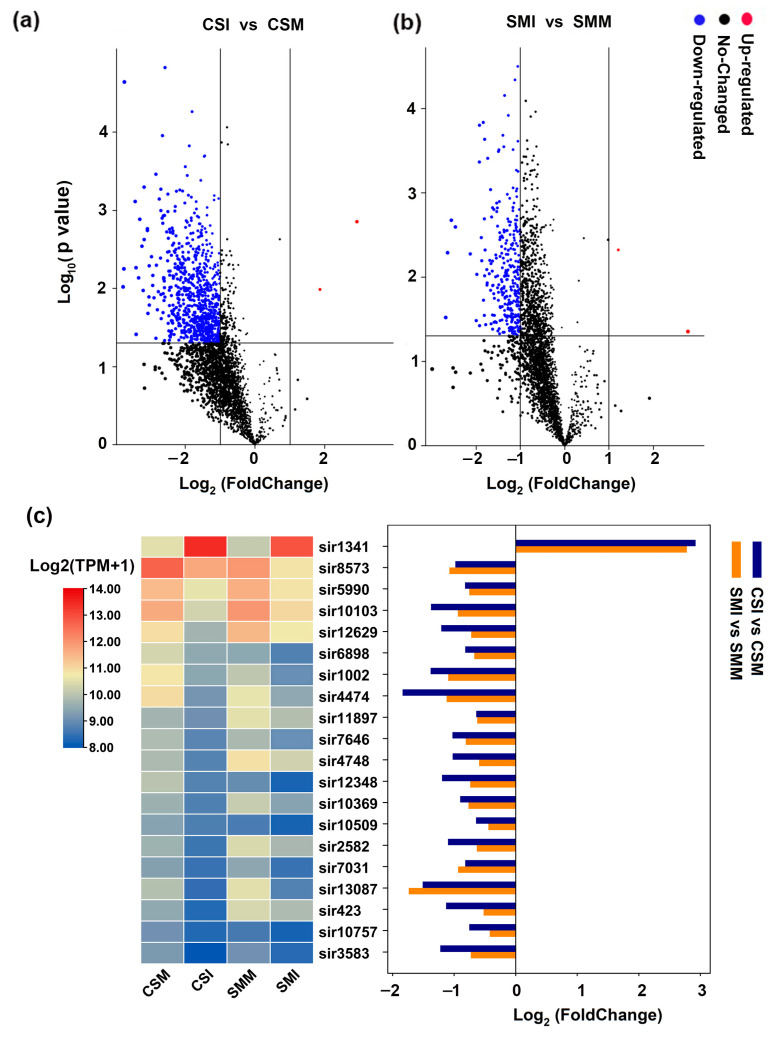
Differential expressions of siRNA. (**a**) Differential expression of siRNA between mock and *F. graminearum* infection in CS. (**b**) Differential expression of siRNA between mock and *F. graminearum* infection in SM. (**c**) The expressional heat map and comparisons of siRNAs with expression abundances being more than 200 TPM. “FoldChange” in the X-axis indicates the multiple of difference, “Negative” represents down-regulation. The Y-axis represents the *p*-value in the biological repetitions. Each dot represents an siRNA. The red dots represent significantly up-regulated siRNA, the blue dots represent significantly down-regulated siRNA and the black dots represent siRNA with no significant difference. The threshold of the difference was set as |Log_2_ (FC)| ≥ 1. The significance difference was analyzed by *t*-test, and the threshold of significance was 0.05.

A total of 3071 siRNAs were co-expressed in CSI, CSM, SMM and SMI. In CS, 2948 siRNA were down-regulated after infection with *F. graminearum*, 114 siRNA were up-regulated, and nine siRNAs had no significant difference between mock and *F. graminearum* infection. When the threshold of difference was set as |Log_2_ (FC)| ≥ 1 and *p* < 0.05, a total of 864 siRNAs were differentially expressed, including 862 down-regulated siRNAs and two up-regulated siRNAs (Figure 2a). In SM, 2819 siRNAs were down-regulated after infection with *F. graminearum*, 244 siRNAs were up-regulated and eight siRNAs had no significant difference between mock and *F. graminearum* infection. When the threshold of difference was set as |Log_2_ (FC)| ≥ 1 and *p* < 0.05, a total of 311 siRNAs were differentially expressed.

Including 309 down-regulated siRNAs and two up-regulated siRNAs (Figure 2b). Twenty siRNAs were highly expressed and more than 200 TPM in each group, among which sir1341 was significantly up-regulated after *F. graminearum* infection and had the highest expression level. The other 19 siRNAs were highly expressed but significantly down-regulated after *F. graminearum* infection (Figure 2c). These results showed that the expressions of siRNAs showed a trend of overall down-regulation after *F. graminearum* infection both in CS and SM.

### 2.3. More siRNAs Were Enriched in Sumai 3 Than in CS

**Figure 3:**
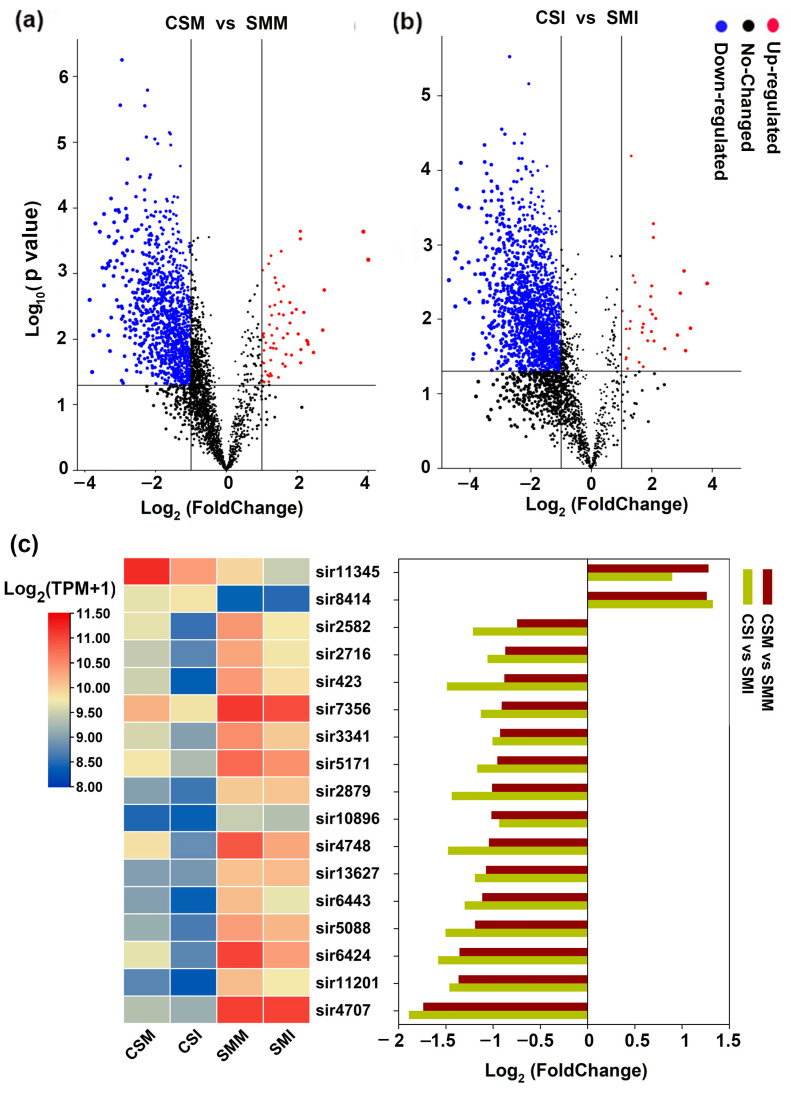
Differential expression of siRNAs among CS and SM. (**a**) Differentially expressed siRNAs between CS and SM under mock inoculation. (**b**) Differentially expressed siRNAs between CS and SM after *F. graminearum* infection. (**c**) Heat map of siRNAs expressions with abundance more than 200 TPM and comparisons between varieties. “FoldChange” in the X-axis indicates the multiple comparison of difference, “Negative” represents down regulation. The Y-axis represents the *p*-value in the biological repetitions. Each dot represents an siRNA. The red dots represent significantly up-regulated siRNA, the blue dots represent significantly down-regulated siRNA and the black dots represent siRNA with no significant difference. The threshold of the difference was set to |Log_2_ (FC)| ≥ 1. The difference in significance was analyzed by *t*-test, and the threshold of significance was 0.05.

In order to compare the differences of siRNAs between CS and SM, the expression patterns of the co-expressed 3071 siRNAs between the two varieties were analyzed. Under mock inoculation, the expression levels of 1170 siRNAs in SM were two or more times higher than those in CS. In comparison, only 63 siRNAs in CS had more than twice the expression levels of those in SM (Figure 3a).

After infection with *F. graminearum*, 1659 siRNA in SM had two or more times the intensity than those in CS, and only 34 siRNAs in CS were higher than those in SM by twice or more (Figure 3b). Among these siRNAs, there were 17 siRNAs with an abundance of more than 200 TPM in each group. sir11345 and sir8414 were highly enriched in CS, and the remaining 15 siRNAs were highly enriched in SM, and three out of 15 siRNAs, sir4748, sir2582 and sir423 showed significant down-regulation after infection with *F. graminearum* (Figure 2c). In summary, more siRNAs were enriched in SM than in CS both in mock inoculation and *F. graminearum* infection.

**Figure 4:**
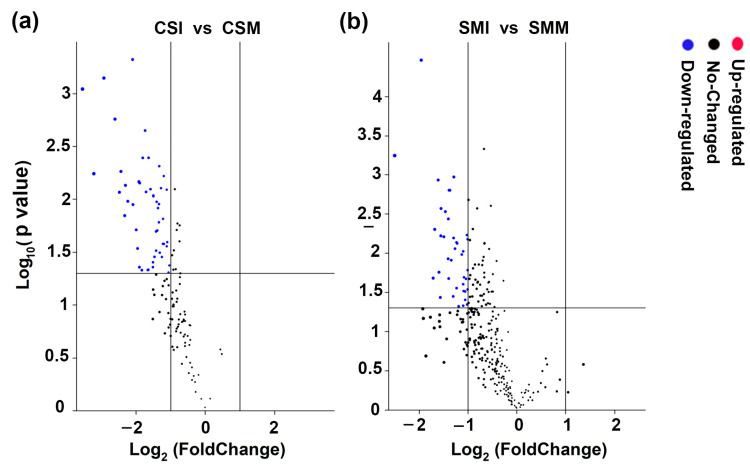
Differential expressions of variety-specific siRNA. (**a**) Differential expressions of CS-specific siRNAs between mock inoculation and *F. graminearum* infection. (**b**) Differential expression of SM-specific siRNAs between mock inoculation and *F. graminearum* infection. “FoldChange” in the X-axis indicates the multiple comparisons of difference, “Negative” represents down regulation. The Y-axis represents the *p*-value in the biological repetitions. Each dot represents an siRNA, The red dots represent significantly up-regulated siRNAs, the blue dots represent significantly down-regulated siRNAs and the black dots represent siRNA with no significant difference. The threshold of the difference was set to |Log_2_ (FC)| ≥ 1. The significance difference was analyzed by *t*-test, and the threshold of significance was 0.05.

In addition, the differences of siRNAs specifically expressed in CS or SM were analyzed. Among the 131 siRNAs specifically expressed in CS (Figure 1d), 128 siRNAs were down-regulated and three siRNAs were up-regulated after *F. graminearum* infection, and 53 out of 128 siRNAs were significantly down-regulated (Figure 4a). Among the 309 siRNAs specifically expressed in SM (Figure 1d), 281 siRNAs were down-regulated, and 28 siRNAs were up-regulated after *F. graminearum* infection, and 42 out of 281 siRNAs were significantly down-regulated (Figure 4b).

Among the CS-specific siRNAs, three siRNAs were silenced after *F. graminearum* infection, and 25 siRNAs were highly significantly down-regulated (*p* < 0.01), and their lengths were mainly 24 nt in length (Table 1). Among the SM-specific siRNAs, two siRNAs were not expressed after *F. graminearum* infection, twenty-two siRNAs were highly significantly down-regulated (*p* < 0.01), and they were mainly 24 nt in length as well (Table 2).

### 2.4. The Expression of TaDCL3 Genes Were Repressed by F. graminearum

**Figure 5:**
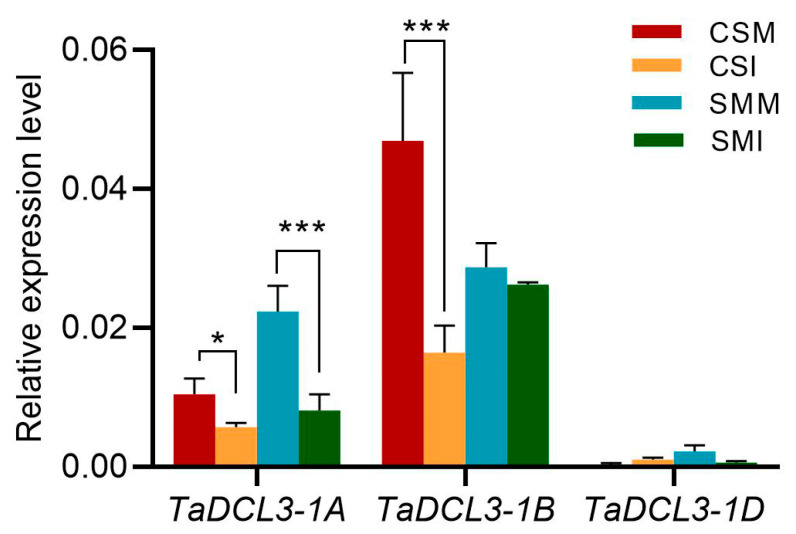
The expression levels of *TaDCL3* genes. Expression levels are shown as mean ± standard error of three biological replicates. Student-*t* test was used for difference analysis, * *p* < 0.05; *** *p* < 0.001.

According to the results above, 24 nt siRNA was the main type of siRNA with the highest expression abundance, and DCL3 was the main ribonuclease used to produce 24 nt siRNA. The homologous genes of DCL3 in wheat have three copies located on the A, B and D sub-chromosomes, which are *TraesCS1A02G160900* (*TaDCL3-1A*), *TraesCS1B02G177600* (*TaDCL3-1B*) and *TraesCS1D02G158000* (*TaDCL3-1D*), respectively. The expressions of siRNAs were overall down-regulated after infection with *F. graminearum* indicating that the synthesis of siRNA may be inhibited. The expressions of *TaDCL3* genes were then analyzed. The results showed that the expression level of *TaDCL3-1D* was extremely low, and the expression level of *TaDCL3-1B* was the highest in each treatment group, followed by the expression level of *TaDCL3-1A*. *TaDCL3-1A* was significantly inhibited by *F. graminearum* both in CS and SM. *TaDCL3-1B* was highly expressed in CS and significantly inhibited by *F. graminearum*, but it was not significantly changed in SM after *F. graminearum* infection (Figure 5). This result indicates that *TaDCL3-1A* and *TaDCL3-1B* might have functional redundancy, but they showed expression differences in the two wheat varieties, and the overall expression pattern of *TaDCL3* is consistent with siRNAs, which implies that the down-regulation of siRNA maybe mainly due to suppression of *TaDCL3* by *F. graminearum*.

### 2.5. Disease Resistance-Related Genes Were Up-Regulated after F. graminearum Infection

**Figure 6:**
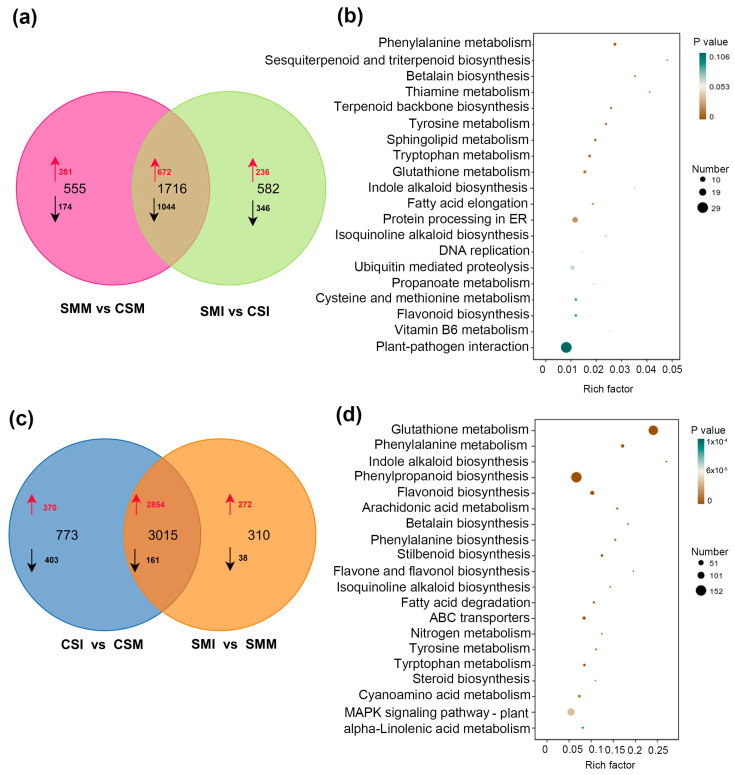
KEGG enrichment of differentially expressed genes between SM and CS. (**a**) Venn graph of differentially expressed genes between mock inoculation and *F. graminearum* infection for SM and CS. “SMM vs. CSM” represents the differentially expressed gene set of SM versus CS under mock inoculation. “SMI vs. CSI” represents the differentially expressed genes set of SM versus CS under *F. graminearum* infection. In the Venn graph, the larger black numbers represent the total number of differential genes; the red arrows and smaller red numbers represent the number of genes whose expression level in SM is significantly higher than those in CS; and the black arrows and smaller black numbers represent the number of genes whose expression level in SM were significantly lower than those in CS. Threshold of the difference was set to |Log_2_ (FC)| ≥ 2. (**b**) KEGG pathway enrichment bubble chart of 1218 significantly lower genes in SM than those in CS under mock inoculation. (**c**) Venn diagram of differentially expressed genes between SM and CS after mock inoculation and *F. graminearum* infection. “CSI vs. CSM” represents the differentially expressed genes set of CS with *F. graminearum* infection versus CS with mock inoculation; “SMI vs. SMM” represents the differentially expressed gene set of SM with *F. graminearum* infection versus SM with mock inoculation. In Venn graph, the larger black numbers represent the number of differential genes, the red arrows and smaller red numbers represent the number of up-regulated genes, and the black arrows and smaller black numbers represent the number of down-regulated genes. Threshold of the difference was set to |Log_2_ (FC)| ≥ 2. (**d**) KEGG pathway enrichment bubble chart of 3126 significantly upregulated genes in SM under *F. graminearum* infection.

The 24 nt siRNAs mainly mediate the silencing of transposons and genes through RdDM. Our aim was to understand if the differential expressions of siRNAs between the two varieties and between *F. graminearum* infection and mock inoculation were associated with the expressions of functional genes, especially the expressions of plant disease resistance-related genes. We performed transcriptome sequencing to identify genome-wide expression changes of genes. Genome-wide gene expression profiling revealed 108,547 expressed genes, of which 57,472 genes were more than 10 TPM in expression level. Firstly, we compared the differentially expressed genes between CS and SM. At the threshold of 4-fold difference (|Log_2_ (FC)| ≥ 2), regardless of *F. graminearum* infection or mock inoculation, 1716 genes differed between SM and CS. Under mock inoculation, 672 genes were highly expressed in SM, 1044 genes were highly expressed in CS and 381 genes were specifically highly expressed in SM and 174 genes in CS. After *F. graminearum* infection, 236 genes were highly expressed in SM and 346 in CS (Figure 6a). Although the number of siRNAs in SM were significantly higher than those in CS under mock inoculation (Figure 1b), the transcriptome data showed that the number of highly expressed genes in SM (1053) was less than that of CS (1218). The trend of the differentially expressed genes (1218) was opposite to that of siRNA, indicating that the high accumulation of siRNA in SM may lead to the inhibition of the expression of their target genes. Then, KEGG enrichment analysis was performed on the 1218 genes with low expressions in SM which showed that, among the top 20 highly enriched signaling pathways, the most significantly enriched signaling pathway is plant–pathogen interaction pathway (Figure 6b).

The results above showed that siRNAs were overall down-regulated both in SM and CS after the *F. graminearum* challenge. According to the principle of negative regulation between siRNA and their targets, there should be a large number of genes that will be significantly upregulated in SM and CS after infection with *F. graminearum*. We further analyzed the differentially expressed genes after inoculation, and 2854 genes were co-upregulated both in SM and CS, while only 161 genes were down-regulated after being infected with *F. graminearum*. There were 272 genes specifically up-regulated in SM after *F. graminearum* infection, while only 38 genes were down-regulated; 370 genes were specifically up-regulated in CS and 403 genes were down-regulated after infection (Figure 6c). In total, the number of up-regulated genes in the two varieties after infection was five times more than the number of down-regulated genes. In particular, the number of up-regulated genes in SM accounted for 94% of the total differentially expressed genes (Figure 6c), which represents a clear negative correlation with siRNA that were significantly down-regulated after *F. graminearum* infection. KEGG enrichment analysis of the 3126 up-regulated genes in SM after inoculation showed that they were mainly enriched in the glutathione metabolism pathway, phenylpropanoid biosynthesis pathway and plant mitogen-activated protein kinase (MAPK) signaling pathway (Figure 6d). These pathways were proved to be closely related to the plant response to biotic stress.

## 3. Discussion

### 3.1. Inhibition of TaDCL3 by F. graminearum Is the Main Reason for Downregulation of siRNA

Plant sRNA can be changed in response to pathogen invasion, thereby regulate gene expression levels by inducing positive regulators of immune responses [26]. Our previous studies demonstrated that wheat miRNA, siRNA and tRF showed different expression patterns after infection with *F. graminearum*. Among the three types of small RNA, siRNA was the richest small RNA in the FHB-resistant variety of SM [27]. We therefore speculated that siRNA may be closely related to FHB resistance. Indeed, siRNAs were inhibited by *F. graminearum* (Figure 1b and Figure 2) in this study. The more severe the symptoms, the more siRNAs were down-regulated. The positive correlation between the expression patterns of siRNA and TaDCL3 after *F. graminearum* infection (Figure 2 and Figure 5) implied that the inhibition of *TaDCL3* subsequently affects the processing of siRNA pre-cursors, resulting in mature siRNA decrease. In addition, many studies have shown that environmental conditions also have an impact on the cleavage activity of DCL. For example, the activity of DCL could be inhibited by low temperatures, which can decrease the production of siRNAs to block the plant defense against viruses using the RNA-silencing pathway [28]; however, whether biotic stress can also inhibit the activity of DCL is still unclear. Therefore, it is still necessary to continue to study how the expression of *TaDCL3* and the enzyme activity are regulated by *F. graminearum* infection.

### 3.2. Inhibition of siRNA Released Resistance-Related Genes Thereby Improving the Wheat Resistance to FHB

DNA methylation can lead to the inhibition of gene expression, and a 24 nt siRNA silences target genes by the way of RNA-mediated DNA methylation (RdDM) [18]. Coincidentally, 24 nt siRNAs are the main type among the down-regulated siRNAs after *F. graminearum* infection (Figure 1c), which implies that the up-regulation of disease resistance genes might be mainly due to the inhibition of 24 nt siRNAs, thereby inducing the plant disease resistance defense. According to the transcriptome data, the number of significantly up-regulated genes after infection accounted for more than 85% of the total number of differentially expressed genes (Figure 6c), and the number of significantly up-regulated genes after infection in SM accounted for about 95% of the total number of differential genes; additionally, most of these genes are related to the plant disease resistance signaling pathway (Figure 6d). Importantly, the overall up-regulation of functional genes after infection represents an obvious negative correlation with the overall down-regulation of siRNAs. The results suggested that the inhibition of siRNA might disinhibit resistance-related genes, which may be the regulation mechanism of wheat immunity to *F. graminearum* infection. However, whether these significantly up-regulated genes are the target genes directly regulated by the down-regulated siRNA through RdDM needs further verification.

### 3.3. Potential Application of siRNA into Breeding of FHB-Resistance Wheat

In addition to siRNA regulation of the expression of plant genes, siRNAs can also regulate the gene expression of pathogenic microorganisms through the cross-kingdom pathway, thereby inhibiting the pathogenicity of pathogenic bacteria to protect the plant from pathogen infection [25]. In this study, SM contained more siRNAs than CS even when it was not infected by *F. graminearum* (Figure 1b). These highly expressed siRNAs might also be delivered to *F. graminearum* after infection, thereby inhibiting the pathogenicity of *F. graminearum*, which may confer the SM a higher level of wheat resistance to FHB. These potential cross-kingdom siRNAs can be used as candidate siRNAs for host-induced gene silencing (HIGS) technology. Those siRNAs that target wheat-derived resistance-related genes can be silenced through gene editing, thereby improving wheat disease resistance.

*F. graminearum* can absorb dsRNA from the environment, which is then processed into sRNA and induces gene silencing of pathogens with complementary sequences [29]. Therefore, siRNAs are also developed as biopesticides to protect plants from pathogens by spraying an RNA known as spray-induced gene silencing (SIGS) [30]. At present, sRNA has been studied as an approach of biological control of wheat FHB in wheat. For example, microRNA (miR1023) was reported to be cross-kingdom in *F. graminearum* to silence the target gene FGSG_03101, and thereby inhibit the invasion of *F. graminearum* [31]. Therefore, this study provides additional clues for the function of siRNAs in FHB resistance, and also provides more candidate genes that can be used for the biological control of FHB in wheat.

## 4. Materials and Methods

### 4.1. Plant Materials and F. graminearum Inoculation

“Sumai3 (SM)” is a famous FHB-resistant variety carrying *Fhb1* [32,33], and “Chinese Spring (CS)” is a well-known FHB-susceptible variety [34,35]. The grains were sown in the experimental field of Yangzhou University. At the jointing stage, the young seedlings were moved into a condition-controlled phytotron under 24 °C and 16 h of light/8 h of dark cycle. Macroconidial spores of *F. graminearum* strain PH-1were produced in mung bean broth following Bai et al. [36].

At early anthesis of spikes, seven spikes of each variety were inoculated by injecting 10 μL of the spore suspension (100 conidia μL^−1^) into the bilateral forest of the ninth spikelet from the bottom of a spike. Then, 10 μL of mung bean broth was used to mock inoculation as a control. The temperature of the phytotron was set at 28 °C for *F. graminearum* invasion. The inoculated spikelets and their adjoined rachis were collected at 1 day(d), 2 d, 3 d, 4 d, 5 d, 6 d and 7 d post *F. graminearum* (CSI, SMI) and mock inoculations (CSM, SMM), respectively. Three independent biological replicates were conducted.

### 4.2. RNA Extraction

A mixed sample from the seven timepoints was prepared for RNA extraction. Total RNA from the mixed sample was extracted using RNAiso plus reagent (TAKARA BIO INC., Kusatsu, Shiga, Japan) according to the manual instructions. Briefly, the tissue samples were ground, using liquid nitrogen, into powder and about 80 mg of the sample was transferred into 1 mL of precooled RNAiso plus and mixed. Then, 200 μL of chloroform was added into the mixture and centrifuged at 12,000 rpm for 10 min at 4 °C. A total of 700 mL of the supernatant was transferred to a new RNase-free tube and an equal volume of isopropyl alcohol was added for precipitation at −20 °C. After 30 min, the mixture was centrifuged at 12,000 rpm for 15 min and the supernatant was removed. Then, 1 mL of 75% ethanol was used to wash the precipitate, and the RNA pellet was air-dried and dissolved using 50 μL of DEPC-treated water.

### 4.3. Small RNA and mRNA Library Construction

For the small RNA library, 1 μg total RNA was purified by electrophoretic separation on a 15% urea denaturing polyacrylamide-gel electrophoresis (PAGE). In PAGE, 18–30 nt bands were excised and recovered. Then, the small RNAs were ligated to adenylated 3′ adapters annealed to unique molecular identifiers (UMI), followed by the ligation of 5′ adapters. Subsequently, the adapter-ligated small RNAs were transcribed into cDNA by SuperScript II Reverse Transcriptase Kit (Invitrogen, Temecula, CA, USA) followed by amplification with PCR. The PCR products were purified using a QIAquick Gel Extraction Kit (QIAGEN, Germantown, MD, USA). The final ligated PCR products were sequenced using the BGISEQ-500 platform (BGI-Shenzhen, Shenzhen, China).

For the mRNA library, Oligo(dT)-attached magnetic beads were used to purify mRNA and then they were fragmented into small pieces. The library construction methods were consistent with our previous report [27]. The final library was sequenced using the BGISEQ-500 platform (BGI-Shenzhen, China).

### 4.4. Sequencing Data Analysis

The sequenced raw reads of sRNA libraries were processed to remove low-quality reads and adapter sequences using fastx_toolkit (http://hannonlab.cshl.edu/fastx_toolkit/, accessed on 2 February 2010). The clean reads of 18–29 nt in length were mapped to a structural RNA (ribosomal RNAs, transfer RNAs, small nucleolar RNAs and small nuclear RNAs) database (http://rfam.xfam.org/, accessed on 1 November 2022). In order to analyze the abundance of wheat siRNAs, the unmapped reads were aligned to the *Triticum aestivum* reference genome (EnsemblPlants 57 release, https://plants.ensembl.org/Triticum_aestivum, accessed on 1 July 2023) using Bowtie [37]. Relative abundance of unique wheat siRNAs in each library was normalized to transcript per million reads (TPM).

The raw reads of mRNA libraries were filtered with SOAPnuke (v1.5.2) [38]. The clean reads were mapped to the *Triticum aestivum* reference genome using HISAT2 (v2.0.4) [39]. The expression level of a gene was calculated by RSEM (v1.2.12) [40].

### 4.5. qRT-PCR Validation of TaDCL3

The HiScript II Q RT SuperMix Kit (Vazyme Biotechnology Co., Nanjing, China) was used to obtain cDNA. Quantitative real-time PCR was performed with ChamQ SYBR qPCR Master Mix (Vazyme Biotechnology Co., Nanjing, China). Primers were designed by Primer Premier software (version 5.0). The expression level of *TaActin* (*TraesCS1A02G274400.1*) was used as an internal reference. The primers were listed in Table 3. The relative expression levels of *TaDCL3* were calculated using the 2^−∆∆CT^ method. Each reaction was performed in three replicates.

### 4.6. Statistical Analysis

The graphs in this study were drawn using GraphPad Prism (version 5.0) and SigmaPlot 10.0 (Systat Software) (version 10.0.0.54). One-way ANOVA and student’s *t*-test were performed using IBM SPSS Statistics (version 19.0) (IBM, Armonk, NY, USA). TBtools software (version 2.0) [41] was used to generate a heatmap, volcano plot, Venn graphs and KEGG enrichment analysis.

## 5. Conclusions

In this study, transcriptome sequencing of siRNAs in an FHB-resistant variety of SM and an FHB-susceptible variety of CS under mock inoculation and *F. graminearum* infection showed that siRNA lengths were mainly distributed at 24 nt, regardless of wheat genotype, and approximately 90% of the siRNAs showed a down-regulation trend after infection with *F. graminearum*. The main ribonuclease TaDCL3 forming 24 nt was detected by qRT-PCR, and its expression trend was consistent with that of siRNA, indicating that the main reason for the down-regulation of siRNA after *F. graminearum* infection may be the inhibition of *TaDCL3* expression. Meanwhile, the number of significantly up-regulated genes after inoculation accounted for more than 85% of the total number of differentially expressed genes, which showed a significant negative correlation with siRNA. In particular, the differentially expressed genes were closely related to plant disease defense signaling pathway. Our data suggests that the down-regulation of siRNA may alleviate the inhibition of RdDM to activate resistance-related genes, thereby improving the resistance of wheat to FHB. This study provides useful clues for further functional research of key siRNAs involved in wheat response to FHB. Engineering siRNA-producing and their causal genes as well as their target genes could be useful strategies to improve the level of disease resistance of crops.

## Figures and Tables

**Table 1:** Chinese Spring-specific siRNAs significantly down-regulated after *F. graminearum* infection.

siRNA ID	Length	CSI	CSM	Log2 (CSI/CSM)	*p* Value
sir1316	24	NA	52	NA	0.0005
sir7086	24	NA	56	NA	0.0063
sir13238	21	NA	69	NA	0.0023
sir65	24	19	104	−2.48	0.0086
sir422	24	97	275	−1.49	0.0093
sir11652	24	80	173	−1.12	0.0081
sir14089	24	7	78	−3.54	0.0009
sir1202	24	33	104	−1.64	0.0040
sir2890	24	77	188	−1.28	0.0078
sir4944	24	15	93	−2.61	0.0017
sir5351	24	21	192	−3.22	0.0057
sir11759	24	76	409	−2.43	0.0054
sir11357	24	25	62	−1.33	0.0049
sir4897	24	84	237	−1.50	0.0092
sir14609	24	16	79	−2.30	0.0074
sir195	24	14	52	−1.89	0.0070
sir987	24	17	57	−1.74	0.0022
sir6137	21	47	108	−1.20	0.0060
sir655	24	158	675	−2.1	0.0005
sir133	24	58	174	−1.58	0.0080
sir13864	24	31	117	−1.92	0.0069
sir1199	24	368	1205	−1.71	0.0085
sir5670	24	74	259	−1.81	0.0041
sir10341	24	7	51	−2.93	0.0007
sir12766	22	37	141	−1.92	0.0068

TPM values in the CSI and CSM columns are the average value over three replicates. TPM: transcripts per million sequences; Log_2_ (Fold Change) represents the multiple of difference between the two groups. NA represents the reads that were not detected.

**Table 2:** Sumai 3-specific siRNAs significantly down-regulated after *F. graminearum* infection.

siRNA ID	Length	SMI	SMM	Log_2_ (SMI/SMM)	*p* Value
sir13416	23	NA	52	NA	0.0001
sir7183	24	NA	56	NA	0.0001
sir2434	24	22	59	−1.39	0.0016
sir6352	24	103	254	−1.29	0.0064
sir4847	24	17	51	−1.56	0.0027
sir14240	23	23	56	−1.27	0.00877
sir6683	24	21	63	−1.62	0.0012
sir2749	24	63	356	−2.51	0.0006
sir7740	24	33	79	−1.24	0.0073
sir12008	24	33	78	−1.23	0.0075
sir4210	24	29	93	−1.69	0.0049
sir1979	24	39	102	−1.37	0.0016
sir8479	23	81	163	−1.01	0.0066
sir10710	24	59	120	−1.03	0.0059
sir9799	24	31	81	−1.41	0.0036
sir10021	24	627	1346	−1.10	0.0095
sir235	24	26	71	−1.47	0.0029
sir7489	21	108	265	−1.29	0.0011
sir14172	24	21	62	−1.56	0.0059
sir6136	21	35	98	−1.49	0.0062
sir1594	21	46	179	−1.96	0.0001
sir12405	22	92	227	−1.29	0.0011

TPM values in the SMI and SMM columns are the average value over three replicates. TPM: transcripts per million sequences; Log_2_ (Fold Change) represents the multiple of difference between the two groups. NA represents the reads that were not detected.

**Table 3:** Primers for qRT-PCR.

Name	Forward	Reverse
*TaDLC3-1A*	CGGCTCAAAATGGACAAAGG	TCAGCGATGCTGAATCCTGG
*TaDLC3-1B*	AACTTCTCGGTCAAGGGCCT	TATTGCACCGGCAATGCTTT
*TaDLC3-1D*	GTCATTTTCCTCCCCCCAAAC	GGAAGCGCATGTCTGTAGGC
*TaActin*	ACCTTCAGTTGCCCAGCAAT	CAGAGTCGAGCACAATACCAGTTG

## Data Availability

All sequences generated by sequencing in this study can be found in the NCBI Short Reads Archive (SRA) BioProject PRJNA683746 (https://www.ncbi.nlm.nih.gov/sra/?term=PRJNA683746, accessed on 10 December 2020).

## Supplementary Materials

The following supporting information can be downloaded at: https://www.mdpi.com/article/10.3390/ijms242116005/s1.

## References

[1] Alisaac E., Mahlein A.-K. (2023). Fusarium head blight on wheat: Biology, modern detection and diagnosis and integrated disease management. Toxins.

[2] Chen Y., Kistler H.C., Ma Z. (2019). *Fusarium graminearum* trichothecene mycotoxins: Biosynthesis, regulation, and management. Annu. Rev. Phytopathol..

[3] McMullen M., Jones R., Gallenberg D. (1997). Scab of wheat and barley: A re-emerging disease of devastating impact. Plant Dis..

[4] Walter S., Nicholson P., Doohan F.M. (2010). Action and reaction of host and pathogen during Fusarium head blight disease. New Phytol..

[5] Vogelgsang S., Hecker A., Musa T., Dorn B., Forrer H.R. (2011). On-farm experiments over 5 years in a grain maize/winter wheat rotation: Effect of maize residue treatments on *Fusarium graminearum* infection and deoxynivalenol contamination in wheat. Mycotoxin Res..

[6] Bai G., Shaner G. (2004). Management and resistance in wheat and barley to Fusarium head blight. Annu. Rev. Phytopathol..

[7] Buerstmayr H., Ban T., Anderson J.A. (2008). QTL mapping and marker assisted selection for *Fusarium* head blight resistance in wheat. Cereal Res. Commun..

[8] Cai J., Wang S., Su Z., Li T., Zhang X., Bai G. (2019). Meta-analysis of QTL for *Fusarium* head blight resistance in Chinese wheat landraces. Crop J..

[9] Zheng T., Hua C., Li L., Sun Z., Yuan M., Bai G., Humphreys G., Li T. (2020). Integration of meta-QTL discovery with omics: Towards a molecular breeding platform for improving wheat resistance to *Fusarium* head blight. Crop J..

[10] Waldron B.L., Moreno-Sevilla B., Anderson J.A., Stack R.W., Frohberg R.C. (1999). RFLP Mapping of QTL for *Fusarium* head blight resistance in wheat. Crop Sci..

[11] Schweiger W., Steiner B., Vautrin S., Nussbaumer T., Siegwart G., Zamini M., Jungreithmeier F., Gratl V., Lemmens M., Mayer K.F. (2016). Suppressed recombination and unique candidate genes in the divergent haplotype encoding Fhb1, a major *Fusarium* head blight resistance locus in wheat. Theor. Appl. Genet..

[12] Li T., Zhang H., Huang Y., Su Z., Deng Y., Liu H., Mai C., Yu G., Li H., Yu L. (2019). Effects of the *Fhb1* gene on *Fusarium* head blight resistance and agronomic traits of winter wheat. Crop J..

[13] Ma X., Liu C., Cao X. (2021). Plant transfer RNA-derived fragments: Biogenesis and functions. J. Integr. Plant Biol..

[14] Borges F., Martienssen R.A. (2015). The expanding world of small RNAs in plants. Nat. Rev. Mol. Cell Bio..

[15] Tang G. (2005). siRNA and miRNA: An insight into RISCs. Trends Biochem. Sci..

[16] Wang Q., Xue Y., Zhang L., Zhong Z., Feng S., Wang C., Xiao L., Yang Z., Harris C.J., Wu Z. (2021). Mechanism of siRNA production by a plant Dicer-RNA complex in dicing-competent conformation. Science.

[17] Dalmay T., Hamilton A., Rudd S., Angell S., Baulcombe D.C. (2000). An RNA-Dependent RNA polymerase gene in Arabidopsis is required for posttranscriptional gene silencing mediated by a transgene but not by a virus. Cell.

[18] Zhan J., Meyers B. (2023). Plant small RNAs: Their biogenesis, regulatory roles, and functions. Annu. Rev. Plant Biol..

[19] Song X., Li Y., Cao X., Qi Y. (2019). MicroRNAs and their regulatory roles in plant-environment interactions. Annu. Rev. Plant Biol..

[20] Wu H., Li B., Iwakawa H.O., Pan Y., Tang X., Ling-Hu Q., Liu Y., Sheng S., Feng L., Zhang H. (2020). Plant 22-nt siRNAs mediate translational repression and stress adaptation. Nature.

[21] Henderson I.R., Zhang X., Lu C., Johnson L., Meyers B.C., Green P.J., Jacobsen S.E. (2006). Dissecting Arabidopsis thaliana DICER function in small RNA processing, gene silencing and DNA methylation patterning. Nat. Genet..

[22] Xie Z., Allen E., Wilken A., Carrington J.C. (2005). DICER-LIKE 4 functions in trans-acting small interfering RNA biogenesis and vegetative phase change in *Arabidopsis thaliana*. Proc. Natl. Acad. Sci. USA.

[23] Katiyar-Agarwal S., Morgan R., Dahlbeck D., Borsani O., Villegas A., Zhu J.K., Staskawicz B.J., Jin H. (2006). A pathogen-inducible endogenous siRNA in plant immunity. Proc. Natl. Acad. Sci. USA.

[24] Colombo M., Raposo G., Thery C. (2014). Biogenesis, secretion, and intercellular interactions of exosomes and other extracellular vesicles. Annu. Rev. Cell Dev. Biol..

[25] Hou Y., Zhai Y., Feng L., Karimi H.Z., Rutter B.D., Zeng L., Choi D.S., Zhang B., Gu W., Chen X. (2019). A phytophthora effector suppresses Trans-Kingdom RNAi to promote disease susceptibility. Cell Host Microbe.

[26] Kamthan A., Chaudhuri A., Kamthan M., Datta A. (2015). Small RNAs in plants: Recent development and application for crop improvement. Front. Plant Sci..

[27] Sun Z., Hu Y., Zhou Y., Jiang N., Hu S., Li L., Li T. (2022). tRNA-derived fragments from wheat are potentially involved in susceptibility to Fusarium head blight. BMC Plant Biol..

[28] Szittya G., Silhavy D., Molnar A., Havelda Z., Lovas A., Lakatos L., Banfalvi Z., Burgyan J. (2003). Low temperature inhibits RNA silencing-mediated defence by the control of siRNA generation. EMBO J..

[29] Qiao L., Lan C., Capriotti L., Ah-Fong A., Nino Sanchez J., Hamby R., Heller J., Zhao H., Glass N.L., Judelson H.S. (2021). Spray-induced gene silencing for disease control is dependent on the efficiency of pathogen RNA uptake. Plant Biotechnol. J..

[30] Werner B.T., Gaffar F.Y., Schuemann J., Biedenkopf D., Koch A.M. (2020). RNA-Spray-Mediated silencing of *Fusarium graminearum* AGO and DCL genes improve barley disease resistance. Front. Plant Sci..

[31] Jiao J., Peng D. (2018). Wheat microRNA1023 suppresses invasion of *Fusarium graminearum* via targeting and silencing *FGSG_03101*. J. Plant Interact..

[32] Zhou M.P., Hayden M.J., Zhang Z.Y., Lu W.Z., Ma H.X. (2010). Saturation and mapping of a major Fusarium head blight resistance QTL on chromosome 3BS of Sumai 3 wheat. J. Appl. Genet..

[33] Schweiger W., Steiner B., Ametz C., Siegwart G., Wiesenberger G., Berthiller F., Lemmens M., Jia H., Adam G., Muehlbauer G.J. (2013). Transcriptomic characterization of two major Fusarium resistance quantitative trait loci (QTLs), *Fhb1* and *Qfhs.ifa-5A*, identifies novel candidate genes. Mol. Plant Pathol..

[34] Choulet F., Wicker T., Rustenholz C., Paux E., Salse J., Leroy P., Schlub S., Le Paslier M.C., Magdelenat G., Gonthier C. (2010). Megabase level sequencing reveals contrasted organization and evolution patterns of the wheat gene and transposable element spaces. Plant Cell.

[35] Ma H.X., Bai G.H., Zhang X., Lu W.Z. (2006). Main effects, epistasis, and environmental interactions of quantitative trait Loci for Fusarium head blight resistance in a recombinant inbred population. Phytopathology.

[36] Bai G., Kolb F.L., Shaner G., Domier L.L. (1999). Amplified fragment length polymorphism markers linked to a major quantitative trait locus controlling scab resistance in wheat. Phytopathology.

[37] Langmead B., Trapnell C., Pop M., Salzberg S.L. (2009). Ultrafast and memory-efficient alignment of short DNA sequences to the human genome. Genome Biol..

[38] Li R., Li Y., Kristiansen K., Wang J. (2008). SOAP: Short oligonucleotide alignment program. Bioinformatics.

[39] Kim D., Langmead B., Salzberg S.L. (2015). HISAT: A fast spliced aligner with low memory requirements. Nat. Methods.

[40] Li B., Dewey C.N. (2011). RSEM: Accurate transcript quantification from RNA-Seq data with or without a reference genome. BMC Bioinform..

[41] Chen C., Chen H., Zhang Y., Thomas H.R., Frank M.H., He Y., Xia R. (2020). TBtools: An integrative toolkit developed for interactive analyses of big biological data. Mol. Plant.

